# Gender Ideologies in Europe: A Multidimensional Framework

**DOI:** 10.1111/jomf.12453

**Published:** 2018-01-11

**Authors:** Daniela Grunow, Katia Begall, Sandra Buchler

**Affiliations:** ^1^ Goethe‐University Frankfurt am Main; ^2^ Utrecht University

**Keywords:** cultural diversity, family policy, gender, gender roles, measurement, quantitative methodology

## Abstract

The authors argue, in line with recent research, that operationalizing gender ideology as a unidimensional construct ranging from traditional to egalitarian is problematic and propose an alternative framework that takes the multidimensionality of gender ideologies into account. Using latent class analysis, they operationalize their gender ideology framework based on data from the 2008 European Values Study, of which eight European countries reflecting the spectrum of current work–family policies were selected. The authors examine the form in which gender ideologies cluster in the various countries. Five ideology profiles were identified: egalitarian, egalitarian essentialism, intensive parenting, moderate traditional, and traditional. The five ideology profiles were found in all countries, but with pronounced variation in size. Ideologies mixing gender essentialist and egalitarian views appear to have replaced traditional ideologies, even in countries offering some institutional support for gendered separate spheres.

Despite rising rates of female employment, European societies are currently promoting different ideals regarding how men and women should divide paid and unpaid work. These ideals are on the one hand reflected in work–family policies, that is, laws and infrastructure supporting women and men as workers and caregivers. On the other hand, they exist in the form of gender ideologies. Gender ideologies characterize joint constructions of meaning and reality in a society and are generally conceptualized as “individuals' levels of support for a division of paid work and family responsibilities that is based on the belief in gendered separate spheres” (Davis & Greenstein, 2009, p. 87). Cross‐national research studying the gender division of paid work and family responsibilities has directed much attention toward welfare states and work–family policies (i.e., Lewis, 1992; Mandel & Semyonov, 2006). Few comparative studies, however, have investigated the composition of gender ideologies within countries and how congruent these ideologies are with existing work–family policy settings (Knight & Brinton, 2017; Pfau‐Effinger, 2012; van Oorschot, Opielka, & Pfau‐Effinger, 2008). The question of correspondence between gender ideologies and work–family policies is of relevance because gender ideologies potentially reinforce or weaken the effects of certain policies (Pfau‐Effinger, 2005). In particular, gender ideologies have been argued to reduce gender inequalities beyond the effect of policies (Budig, Misra, & Böckmann, 2012). The first aim of our study is thus to contribute to filling the gap in knowledge on what gender ideologies actually look like in diverse work–family policy settings in which different work–family arrangements are practiced. Although we are not able to test causal links between gender ideologies and work–family policies, we draw on the recent literature on policy feedback theory (Campbell, 2012; Gangl & Ziefle, 2015) and discuss mechanisms through which country‐level differences in work–family policies and practices may intersect with individual‐level gender ideologies.

Although gender ideology is usually framed as a unidimensional concept ranging from egalitarian to traditional (Davis & Greenstein, 2009, p. 95), we argue that it needs to be conceptualized and empirically assessed as a multidimensional concept because beliefs about the roles of men and women are more complex than a single continuum with traditional at one end and egalitarian at the other. For instance, support for joint family responsibilities may not necessarily coincide with support for joint earning and vice versa. Likewise, policy frameworks may support one and not the other; for example, empirical assessments of all European Union countries show that work–family policies may foster dual earning but limit dual caring (Saraceno & Keck, 2011). Extending these arguments, cross‐national research on gender ideologies suggests that societies can become more egalitarian on one dimension of gender ideology and at the same time more traditional on another (Yu & Lee, 2013). Concomitant inconsistencies in attitudes and divisions of labor have frequently been documented in the United States, Europe, and Australia (England, 2011; Treas & Drobnic, 2010; van Egmond, Baxter, Buchler, & Western, 2010). In these contexts, although mothers and fathers generally both participate in paid work, even during the early stages of family formation divisions of housework and care have remained gendered. Despite this, the majority of studies explicitly examining gender ideologies or employing a measure of gender ideology as a control variable use either one item or create a composite measure or index of various items addressing gender attitudes. Both of these approaches are not ideal due to the loss of information on different dimensions of gender ideologies (Ciabattari, 2001). Thus, the second aim of our study is to propose and provide empirical support for a theoretical framework that takes the multidimensionality of gender ideologies into account.

We propose to fulfill these two aims by assessing (a) whether gender ideologies are unidimensional, as suggested in the separate spheres framework, or rather multidimensional, as is indicated by an increasing body of predominantly qualitative research that finds a spread of ideologies that combine traditional and egalitarian views; (b) whether the prevalence of different gender ideology dimensions varies across countries and between men and women; and (c) whether the prevalence of gender ideologies corresponds with work–family policies. Theoretically, we employ policy feedback theory (Campbell, 2012) to frame comparative literature on work–family policies (i.e., Misra, Budig, & Moller, 2007; Saraceno & Keck, 2011) and to illustrate how country‐level differences in work–family policies and practices intersect with the individual‐level gender ideologies. Empirically, we apply a latent class analysis to allow for diverse gender ideology profiles within and between work–family policy settings. We detect different gender ideology profiles (latent classes) in a sample of eight European countries and predict latent class membership by country and sex of respondent to assess the within‐ and between‐country variations in these classes.

Our article extends research on gender ideology in several ways. First, we use large representative national samples from Europe, drawing on comprehensive gender ideology items to assess the multidimensionality of gender ideologies. Second, we study how widespread these multidimensional ideologies are across the countries we examine. Third, we provide tentative evidence on the question of congruence between work–family policy setting and gender ideology by comparing salient dimensions of both across dissimilar institutional work–policy settings.

## Gender Ideology—Concept and State of Research

Gender ideologies are believed to be complex in nature and constructed over time, both as an individual matures and obtains life experience and also as historical time passes (Davis & Greenstein, 2009, p. 95). On an individual level, both interest‐based and experience‐based factors account for variation in gender ideologies (Bolzendahl & Myers, 2004), with both being influenced by work–family policies and related changes in the gender culture (Gangl & Ziefle, 2015; Pfau‐Effinger, 2012). Our focus in this article is on gender ideologies in the specific domain of family and hence on ideologies concerning the centrality of family, work, and care in men's and women's lives. We first review the prevailing ideologies currently identified in the literature and contrast them in light of the following three dimensions considered salient (Table 1): earning as a gender separate (male) or joint sphere, caring as a gender separate (female) or joint sphere (Davis & Greenstein, 2009), and whether the ideology emphasizes choice or gendered traits (Knight & Brinton, 2017). Second, we relate these dimensions to work–family policies that have been argued to be important markers of country‐level differences in work–family policies and practices (Budig et al., 2012; Saraceno & Keck, 2011) and elaborate on the potential mechanisms that may link country‐level policies and practices with the individual‐level gender ideologies assessed.

**Table 1:** Male, Female, or Joint Spheres: Gendered Work–Care Ideologies and Work–Family Policies

	Earning		Caring		Emphasis on…	
Gender ideologies	Separate male	Joint	Separate female	Joint	Choice	Gendered traits
Unidimensional						
Egalitarian		X		X	X	
Traditional	X		X			X
Multidimensional						
Intensive mothering/parenting	X			X		X
Egalitarian essentialism	X	X	X	X	X	X
Policies					Policy outcome strengthens…	
					Choice	Gendered traits
Parental leave		X		X	X^a^	X^b^
Leave reserved for fathers (paid)		X		X	X	
Maternity leave (paid)^c^		X	X			X
Child care (< age 3)		X			X	

*Note*. Own stylized depiction.

a If well‐paid.

b If unpaid or low paid.

c In most countries, taking maternity leave is mandatory for working mothers. In some countries, the length of maternity leave can be varied, providing some limited element of choice for mothers.

### *Unidimensional Ideologies*

Within the current framework used to assess gender ideologies, strong beliefs in women's and men's dual breadwinner and caregiver roles are usually labeled egalitarian gender ideologies (Ritzer, 2007) or “liberal egalitarianism” (Knight & Brinton, 2017, p. 1487). Egalitarian ideologies reflect a belief in men's and women's joint responsibility and capability for earning and caring and emphasize individual choice, not gendered traits, in regard to the arrangements adopted in practice (Davis & Greenstein, 2009; Orloff, 2008; see Table 1 for a stylized overview). Traditional gender ideologies, in contrast, generally refer to beliefs in gendered separate spheres in the employment and family domains (Davis & Greenstein, 2009; Kroska, 2007). Specifically, individuals holding traditional ideologies consider the sphere of earning as male and separate from the female domain of care and unpaid work at home. Proponents of traditional ideologies do not emphasize choice in which partner should be earning or caring because they consider divisions of labor as resulting from gendered traits (Charles & Bradley, 2009). We refer to this framework of egalitarian and traditional ideologies as unidimensional, as, within this framework, gender ideologies fall along a continuum of joint and separate spheres with limited room for mixed ideologies (e.g., support for joint breadwinning but rejection of joint care). In principle, another possible unidimensional ideology would be reverse traditionalism, but, according to the literature, few individuals would consider earning a separate female sphere and caring a separate male sphere, and role reverse practices appear to reflect adaptations to circumstances, not an embodiment of reverse‐traditional ideals (Girardin, Bühlmann, Hanappi, Le Goff, & Valarino, 2016; Ranson, 2015).

Within the unidimensional framework, different experiences during socialization, education, and paid work have been argued to foster distinct gender ideologies among women and men (Davis & Greenstein, 2009). Given the rise in women's educational and occupational attainment as well as in mothers' labor force participation, one would expect women's gender ideologies to become less supportive of gendered separate spheres. For instance, Goldscheider, Bernhardt, and Lappagård (2015) have argued that the ongoing gender revolution would undermine the structures that have been built around ideologies of separate spheres. Others have argued that men should hold less egalitarian beliefs than women because they expect to gain less from gender equality than women (Bolzendahl & Myers, 2004). In this context, authors hint about efforts to defend an ideology of male supremacy (Connell, 2005), with tensions between earning and caring appearing to limit men's willingness and ability to renegotiate gender roles (Gregory & Milner, 2011). Although men's involvement in care work has increased in particular countries and more among the highly educated (Sullivan, Billari, & Altintas, 2014), the “second half” of the gender revolution is still ongoing, and its pace remains debated (Goldscheider et al., 2015, p. 208). This suggests that a simple egalitarianization of gender ideologies over time is not necessarily a given despite women's increasing labor market attachment.

Indeed, despite research that indicates that both men's and women's attitudes have shifted toward more egalitarian ideologies since the 1970s (Cotter, Hermsen, & Vanneman, 2011; Davis & Greenstein, 2009), with evidence of a slower pace of change for men (Ciabattari, 2001), the increasing trend toward gender equality appears to have stalled in a number of countries, including the United States (England, 2011) and Australia (van Egmond et al., 2010). In addition, research points to the appearance of new gender ideologies that are transverse to the unidimensional egalitarian or traditional axis, referred to here as *multidimensional ideologies* (Knight & Brinton, 2017; Yu & Lee, 2013).

### *Multidimensional Ideologies*

Concurrently, evidence shows that ideologies of intensive mothering (Hays, 1996), intensive parenting (Wall, 2010), and egalitarian essentialism (Charles & Grusky, 2004; Cotter et al., 2011) have been on the rise. We refer to these ideologies as multidimensional because these concepts are difficult to assess empirically on a continuum between egalitarian and traditional.

Intensive mothering places mothers primarily as caregivers and urges them to organize their life solely around the perceived needs of their child. According to Hays (1996), intensive mothering comprises the following three primary elements: (a) the mothers' primary responsibility for child care; (b) motherhood as a “natural,” female trait and “emotionally absorbing” (Hays, 1996, p. 110); and (c) an emphasis on child‐centered approaches to child rearing as children are considered “sacred” (Hays, 1996, p. 122). Extending this concept more explicitly toward men, Wall (2010) emphasized the fact that concomitant demands are also put on men as breadwinners and caregivers to enable this intensive form of care (Gregory & Milner, 2011). The ideologies of intensive mothering and intensive parenting thus contest egalitarian ideals of maternal employment (joint earning), at the same time demanding a certain degree of shared caring between women and men, in particular fathers' involvement in parenting. As indicated in Table 1, ideologies of intensive mothering and parenting thus consider earning primarily a male sphere, whereas care is considered a joint sphere in which the mother holds the natural primacy but the father is also involved (Hays, 1996; Lee, Macvarish, & Bristow, 2010). There is no emphasis on parents choosing their roles, but on natural gendered traits. To be sure, the concepts of intensive parenting and intensive mothering entail further aspects of parenting that go beyond our comparative framework, for instance, the need for parents to provide constant intellectual stimulation of the child, as has been emphasized by Wall (2010). Empirical analyses of the phenomenon suggest that fathers are less intensive parents than mothers (Hays, 1996) and more likely to hold traditional ideas about fathering (Shirani, Henwood, & Coltart, 2012). Despite this, men's roles as fathers have been found to increasingly intensify (Faircloth, 2014a) as men see good fathering as being involved and emotionally present for their children in addition to their earner role. Despite burgeoning research in this field, only two known quantitative studies examining the concepts of intensive parenting exist. Liss, Schiffrin, Mackintosh, Miles‐McLean, and Erchull (2013) and Schiffrin et al. (2014) developed quantitative scales to evaluate intensive parenting ideologies. They found ideologies of intensive parenting to prevail and their scales to be valid and reliable. Despite this, the homogeneity of their samples (attained via a snowball sampling technique using Facebook and parenting blogs, and college students, respectively) limits the generalizability of the results to White, well‐educated, middle‐ to upper‐class residents of the United States. In addition, these studies focused exclusively on measuring intensive parenting ideologies; thus the results do not allow for assessing how widespread intensive parenting ideologies are compared to traditional and egalitarian ideologies.

Another ideology frequently discussed in the literature is egalitarian essentialism (Charles & Grusky, 2004; Cotter et al., 2011; Knight & Brinton, 2017). Egalitarian essentialism is argued to combine feminist affirmations of choice and the conviction that both the work sphere and the care sphere are of equal value and importance, in particular, the positioning of support for stay‐at‐home mothering as a woman's choice and equivalent to the choice of earning (Cotter et al., 2011). The notion of egalitarian essentialism as put forward by Cotter et al. (2011) theoretically draws on the work by Charles and Grusky (2004, p. 27), who have argued that “deeply rooted and widely shared cultural beliefs about gender difference are ideologically compatible with liberal egalitarian norms.” This is a cultural frame that, although to some degree accepting both joint and separate spheres of earning and caring, exalts traditional gendered traits and discounts hierarchical power relations, denying any implications of lower status or power for women (Cotter et al., 2011; see Table 1). In line with this idea, Yu and Lee (2013), using data from 33 countries, and Knight and Brinton (2017), using data from 17 countries, demonstrated empirically that existing individual support for ideologies toward egalitarianism, essentialism, and individual choice resulted in a spread of gender ideology schemas that do not fit the unidimensional egalitarian or traditional axis. Similarly, Yamaguchi (2000) identified three types of gender‐role profiles among Japanese women comprising one traditional and two egalitarian classes, whereby one of the egalitarian classes is in favor of women's paid work and one other is more critical. These studies point to the formation of different versions of egalitarianism. Another multidimensional approach by Aboim (2010, p. 171) combined factor and cluster analysis to identify the following three patterns of conjugal practices: “unequal sharing,” characterized by negative views toward working mothers and low levels of support for both dual breadwinning and men's participation in unpaid work; “familistic unequal,” characterized by even more negative views toward working mothers and a low support for dual breadwinning, but more positive views on men's participation in unpaid work; and a “dual earner/dual career” pattern with positive views on all three indices.

### *A New Framework for the Operationalization of Gender Ideologies*

We argue that the spread of these new types of ideologies in industrialized societies, in addition to differing interests of men and women in a more egalitarian division of paid work and care, leads to an increased inability to empirically locate contemporary gender ideologies within the unidimensional separate spheres framework with traditional at one end of the continuum and egalitarian at the other. Currently, the majority of studies examining gender ideologies explicitly, or employing a measure of gender ideology as a control variable, nevertheless create a composite measure or index of various items addressing distinct gender attitudes (Ciabattari, 2001; Cotter et al., 2011; Cunningham, Beutel, Barber, & Thornton, 2005; Lucier‐Greer & Adler‐Baeder, 2011). This operationalization forces the separate spheres framework onto the data and resulting analysis. In addition to this method losing information on different dimensions of gender attitudes, which have been found to be important (Ciabattari, 2001), many studies employing these types of composite measures report relatively low reliability scores (Amato & Booth, 1995; Baxter, Buchler, Perales, & Western 2015; Greenstein, 1996). If gender ideologies are indeed increasingly multidimensional, low reliability scores may be a result of weaknesses in the theoretical conceptualization of gender ideologies as unidimensional and based on a traditional or egalitarian dichotomy that rests solely on gender separate spheres. Furthermore, the no longer fitting conceptualization of the gendered separate spheres framework with the traditional or egalitarian dichotomy may also be a contributory factor to the lack of conclusive results on how gender ideologies correspond to different work–family policy settings. We elaborate on this aspect in the next section.

### *Interdependencies Between Gender Ideologies and Work–Family Policy*

The assumption that gender ideologies play a crucial role for work*–*family policy making has been present since the early 2000s (for a review, see Lewis, Knijn, Martin, & Ostner, 2008). Still, the limited number of comparative studies that have investigated the composition of gender ideologies within countries and how these ideologies interact with work*–*family policy settings do not provide a clear‐cut finding (Pfau‐Effinger, 2012; van Oorschot et al., 2008). Whereas cross‐national studies have sometimes found gender ideologies and policy frameworks to be correlated (Lück & Hofäcker, 2008), other studies indicated that these correlations were far from perfect (Bauernschuster & Rainer, 2012; Lewis et al., 2008) and that within‐country variation was more pronounced than cross‐national variation (Edlund & Öun, 2016; Jappens & Van Bavel, 2012). In their recent study, Knight and Brinton (2017) provided evidence that distinct gender ideologies spread with uneven prevalence across various regions of Europe. They suggested that the policies adopted in the various regions prioritized different aspects of either joint or separate spheres, leading to this diversity.

None of these comparative studies, however, specified a mechanism through which country‐level differences in work*–*family policies potentially intersect with individual‐level gender ideologies. Although we do not aim to test a causal link between national context and individual ideologies, we suggest that our three‐dimensional framework, comprising either joint or separate earning and caring, with an emphasis on either choice or gendered traits, may serve as a heuristic to assess existing work*–*family policies in Europe and, thus, their congruence with prevalent gender ideologies (see Table 1). Drawing on policy feedback theory (Campbell, 2012), we argue that work*–*family policies may shift the interests, beliefs, and ideologies of individuals and societies at large. In turn, the interests, beliefs, and ideologies held by citizens (and policy makers) feed back into the policy‐making process (Campbell, 2012). This implies that within countries, over time, policies and ideologies should reach a certain degree of congruence, whereas momentary discrepancies may arise from contemporaneous change in either domain. Within the policy feedback framework, the following two mediating mechanisms though which work*–*family policies may affect individual gender ideologies have been identified and tested: role exposure and norm setting (Gangl & Ziefle, 2015).

The role exposure mechanism suggests that the introduction or extension of parental leave, leave reserved for fathers, or maternity leave may affect individual ideologies by changing caregiver roles and practices in a country. Well‐paid parental leave, paternity leave, and leave reserved for fathers, for instance, create options and incentives for joint earning and caring, resulting in higher degrees of choice among parents (Table 1). These policy designs have been labeled “egalitarian” (Ray, Gornick, & Schmitt, 2010, p. 196), pointing to existing links between work*–*family policies and gender ideologies. Unpaid and low‐paid parental leave, although also legally enabling joint earning and caring, in contrast, has been found to underscore gendered traits and traditional family models, as these leaves are almost exclusively taken up by mothers (Ray et al., 2010). Paid maternity leave is mandatory in Europe and only affects mothers' (not fathers') earner and carer roles. On the one hand, maternity leave targets working mothers as (joint) earners; on the other hand it attributes intensive care provision for newborns exclusively to mothers by linking the biological process of giving birth to the primary carer role for an infant (Knijn & Kremer, 1997; Table 1). Furthermore, the provision of early child care (in particular, the age at which children have a right to a day care place) will affect the duration or intensity of role exposure by granting working parents an alternative to exclusive parental care. Accordingly, social norms regarding the duration and intensity of care to be provided by mothers, fathers, or institutions will be affected.

Thus, the role exposure mechanism has been argued to affect the nature of parenthood in a country as parents react to changing economic incentives to claiming care leaves versus sending their child to a child‐care facility (Gangl & Ziefle, 2015). As a result of policy interventions, crucially, whether mothers, fathers, or both parents are targeted by them, certain principles of joint and separate care provision and earning will become the dominant pattern of parents' behavior, thus a social norm, and finally a reference point for the formation of individual gender ideologies (Gangl & Ziefle, 2015; Grunow & Veltkamp, 2016).

The norm‐setting mechanism, in contrast, provides a link between policy and broader social norms owing to the fact that the work*–*family policies in place have been designed to promote specific gendered models of work and care (Gangl & Ziefle, 2015), usually at the expense of alternative models. By promoting certain models of care and not others, work*–*family policies thus reflect broader gender‐specific social norms of balancing parenthood, care, and earning not only in a behavioral but also in a moral sense (Kremer, 2007; Lewis, 1992; Orloff, 1996).

Drawing on policy feedback theory, Gangl and Ziefle (2015) empirically tested the role exposure and norm‐setting mechanisms in Germany and found that the extension of parental leave generated cultural change by influencing individual preferences, ideologies, and behavior. According to this research, policy feedback affects individual gender ideologies in the sense that policies themselves serve as cultural and normative reference points for individuals (see also Grunow & Veltkamp, 2016). In addition, mass opinion and thus dominant gender ideologies have been identified as major obstacles for the implementation of more transformative, gender‐egalitarian work*–*family policies in Europe (Morgan, 2009, p. 48). Furthermore, it has been argued that during processes of social change, several dominant family models may compete, thereby creating a contradictory gender culture within a given context (Pfau‐Effinger & Euler, 2014). Together this research suggests that the association between gender ideology and work*–*family policies is mutually reciprocal and thus linked (see Table 1) although potentially asynchronous.

### *Empirical Expectations*

The state of research points to the coexistence of both unidimensional gender ideologies (based on an egalitarian or traditional axis within the separate spheres framework) and multidimensional gender ideologies, such as intensive parenting and egalitarian essentialism (which do not fit into the egalitarian or traditional axis). In addition, several family models have been argued to compete at any one time. We thus expect to find more than one dominant gender ideology in Europe, including unidimensional and multidimensional ideologies (Hypothesis 1). Following Bolzendahl and Myers (2004), both interest‐based and experience‐based factors should account for variation in gender ideologies between women and men within a given family policy context. Thus, within any given country, we expect women to hold more egalitarian gender ideologies and men to hold more traditional ideologies (Hypothesis 2a). Multidimensional ideologies are more ambivalent with respect to whether men or women benefit more from the corresponding divisions of labor. Gender differences should thus be less pronounced within the multidimensional ideologies than within the unidimensional ideologies (Hypothesis 2b).

The diverse work*–*family policies implemented by European welfare states contain aspects that conceivably both reflect and generate not only unidimensional but also multidimensional ideologies (i.e., Pfau‐Effinger, 2012). Thus, we expect the prevalence of different gender ideologies to vary across national work*–*family policy settings (Hypothesis 3). To account for this variation, we examine the association between gender ideologies and work*–*family policies in the following eight European countries, which offer varying levels of institutional support for egalitarian and traditional divisions of labor: the Czech Republic, western Germany, the Netherlands, Italy, Poland, Spain, Switzerland, and Sweden. These countries represent the current spectrum of work*–*family policies in Europe, ranging from Sweden, a forerunner in egalitarian family policies fostering practices of joint spheres, to Switzerland, a country not offering parental leave, only compensated maternity leave, thereby strengthening caring as a separate female sphere. Between these two ends of the spectrum, the selected countries offer a range of policies that support different aspects of separate and joint spheres and reflect different historical and cultural developments (Ray et al., 2010; Saraceno & Keck, 2011). A detailed comparative overview of the work*–*family policies effective in the eight countries during the year of data collection (2008) can be found in Appendix Table A1. The next section outlines country and policy‐context specific hypotheses that we derive from our theoretical framework outlined in Table 1 and by drawing on policy feedback theory.

Egalitarian gender ideologies should be most widespread in settings where work*–*family policies have consistently favored joint earning and joint caring. Of all the countries included in our analysis, Sweden fulfills this condition most clearly. It combines well‐paid parental leave, including a paid leave quota for fathers, with comparatively short maternity leave and strong state support for nonparental care for children younger than the age of 3 years. In addition, Sweden has followed this egalitarian policy course for many decades. Thus, we expect to find the highest level of support for egalitarian gender ideologies (and low levels of support for competing ideologies) in Sweden (Hypothesis 3a).

Traditional ideologies should prevail in work*–*family policy settings that have a history of strengthening gendered traits through promotion of separate spheres. Such policy measures would include no, low‐paid, or unpaid parental leave; long maternity leave; and a lack of state supported child care for children younger than the age of 3 years. Although Switzerland, the Czech Republic, and Poland fall into this category to some degree, they do not meet all these criteria at once. The Czech Republic and Poland share a socialist history of men's and women's joint earning and thus no strictly separated spheres, and Switzerland has relatively long, well‐paid maternity leave emphasizing a woman's earner and carer roles. As such, we do not expect any of the countries in our sample to show distinct traditional ideologies (Hypothesis 3b).

Multidimensional ideologies should be most widespread in countries where the work*–*family policy framework does not consistently promote either joint spheres or separate spheres. Germany is a borderline case, as, similar to Sweden, it offers well‐paid parental leave, including a paid leave quota for fathers, thus institutionally enabling egalitarian choices and the spread of egalitarian ideologies. However, these policies, introduced in 2007, were very recent in the year when our data were collected (2008). In addition, at the time, state support for child care for children younger than the age of 3 years was low (although rising), and policies supporting separate spheres remained in place, including joint taxation and the option to extend parental leave (without further financial compensation) for up to 3 years after childbirth. Consequently, we expect a strong emergence of egalitarian ideologies in Germany to be competing with traditional and multidimensional ideologies (Hypothesis 3c). The remaining countries in our sample have less clear‐cut work*–*family policies. Although the Netherlands has a tradition of separate spheres, since the 1990s unpaid parental leave has been offered, including an unpaid leave for fathers, with the idea that both mothers and fathers combine part‐time care leaves with part‐time employment. In addition, financial support for early child care is granted. Italy and Spain have been classified as lacking work*–*family policies; they nevertheless offer some elements of potential choice for joint caring (Saraceno & Keck, 2011). In Italy, 6 months of parental leave are reserved for fathers although the paid months are usually taken by the mother right after the maternity leave, the remainder is unpaid and thus rarely used (Organization for Economic Cooperation and Development, 2015). In Spain, fathers have a right to claim 2 weeks of paid paternity leave, which are used by the majority of fathers (at present, 58% claim paternity leave; Eurofound, 2015). Additional parental leave is unpaid, so few fathers use it. As noted previously, the work*–*family policy contexts of Switzerland, the Czech Republic, and Poland do not effectively back either joint or separate spheres. In sum, the Czech Republic, Italy, the Netherlands, Poland, Spain, and Switzerland would be expected to foster a prevalence of competing multidimensional gender ideologies rather than unidimensional gender ideologies (Hypothesis 3d).

In addressing these hypotheses within our theoretical framework, we aim to examine the level of congruence between the work*–*family policies in each country and which particular gender ideologies dominate in each context. The following section outlines our methodological approach.

## Method

### *Data*

The data for our analysis come from the fourth wave of the European Values Study (EVS) collected in 2008 (EVS, 2011). The EVS is a large‐scale international survey focusing on values and attitudes and changes in these over time. A representative multistage stratified random sample of the adult population of each country aged 18 years and older was used for the EVS 2008. The net sample size was 1,500 respondents per country. Fieldwork was conducted on the basis of detailed and uniform instructions provided to all countries by the EVS advisory groups. The EVS questionnaires were administered as face‐to‐face interviews in the appropriate national languages (EVS, 2011). We selected respondents from eight countries (the Czech Republic, Poland, western Germany, Switzerland, Italy, Spain, the Netherlands, and Sweden) aged 18 to 45 years for our analytical sample (*N* = 5,153 respondents, 54% female). The selected age range reflects our theoretical interest in the gender ideologies held by respondents who may be involved in processes of family formation and thus (potentially) affected by country‐specific work–family policies, as these would be the ones to adapt to policy changes first and thus foster policy‐induced cultural change (Gangel & Ziefle, 2015). Data were weighted by population size to make the proportions of respondents from different countries in our sample reflect the real size of these countries (see Appendix Table A2).

### *Measures*

We employ seven items measuring attitudes toward gender ideologies. As a result of the skewed nature of the responses and the absence of a neutral category, the original response categories of these items (1 = “strongly agree,” 2 = “agree,” 3 = “disagree,” 4 = “strongly disagree”) were dichotomized and where necessary reverse‐coded so that 1 reflects (strong) agreement with an egalitarian response and 0 reflects (strong) agreement with a traditional response. We consider an egalitarian response to signify a belief in joint spheres, whereas a traditional response reflects belief in separate spheres (for any given item). In referring to separate or joint spheres, we aim to move away from terminology employed in unidimensional conceptions of gender ideology. We used the following items (see Table 2 and Appendix Figure A4 for the overall agreement with attitudes supporting joint spheres in the sample):

1. A working mother can establish just as warm and secure a relationship with her children as a mother who does not work.
2. A preschool child is likely to suffer if his or her mother works. (reversed)
3. Being a housewife is just as fulfilling as working for pay. (reversed)
4. A job is alright, but what most women really want is a home and children. (reversed)
5. Both the husband and wife should contribute to household income.
6. In general, fathers are as well suited to look after their children as mothers.
7. Men should take as much responsibility as women for the home and children.

**Table 2:** Conditional Probability of Agreement With Egalitarian Gender Ideology and Class Size for the Five Class Model (N = 5,153)

	Egalitarian	Egalitarian essentialism	Intensive parenting	Moderate traditional	Traditional	Sample mean
Class size, %	42.8	22.3	22.0	7.3	5.6	
Items						
1. Agree: Working mother warm relationship with children	.95	.91	.42	.61	.09	.76
2. Disagree: Preschool child suffers with working mother	.80	.47	.01	.37	.00	.47
3. Disagree: Being housewife as fulfilling as paid job	.76	.12	.62	.58	.05	.53
4. Disagree: Job alright, but women really want home & children	.79	.28	.39	.48	.05	.53
5. Agree: Husband & wife should contribute to household income	.92	.83	.91	.57	.42	.84
6. Agree: Fathers as well suited to look after children as mothers	.89	.93	.76	.28	.42	.80
7. Agree: Men should take same responsibility for home & children	.99	.98	.98	.30	.72	.92

*Source*. European Values Study (2011), calculations by authors.

*Note*. Data are weighted by population size. Items 2, 3, and 4 were reverse coded.

### *Analysis*

We employ latent class analysis (LCA) to detect gender ideology profiles in our sample. LCA was developed to classify cases into profiles on the basis of responses given to a set of categorical indicators; these profiles are known as classes and associated class memberships (Lazarsfeld, Henry, & Anderson, 1968). However, contrary to factor analysis, LCA is a person‐centered approach, meaning that the method allows for classifying individuals into distinct classes based on their own individual response patterns. LCA relies on the assumption of local independence, which means that, given class membership, item responses are independent. In other words, it is assumed that the relationship between responses is fully captured by the latent structure. We use Mplus 7.2 (Muthén & Muthén, 1998–2012) to estimate our models. Missing values were treated using full information maximum likelihood estimation. The covariates sex and country of residence were included in the estimation of the latent class structure. The decision of how many classes are appropriate in LCA is guided by both measures of model fit and theoretical considerations and interpretability of the class structure. We estimated seven models comprising one through seven class solutions, with sufficient random starting value perturbations to ensure that the best log‐likelihood was replicated and our solutions did not present local maxima. Model fit was assessed using the Bayesian information criterion and the Lo–Mendell–Rubin likelihood ratio test. In our case, the Lo–Mendell–Rubin likelihood ratio test indicated a five‐class model, but the lowest Bayesian information criterion value was found for the six‐class model. Because the decrease in Bayesian information criterion clearly leveled off after five classes and the five‐class model was distinctly interpretable in light of the state of research, our preferred solution was the five‐class model (fit statistics for all models are presented in Appendix Table A3). To account for violations of the assumption of local independence and measurement invariance, direct effects of covariates on the class indicators were tested and kept in the model when significant. The final model including the significant direct effects shows improved model fit compared to the model without these effects and also outperforms a fully heterogeneous model in which the class structures are allowed to differ between countries. To assess country and sex differences in the final LCA model, we calculated the predicted probabilities of class membership for men and women per country in a multinomial logit model.

To test for the repeatability and robustness of our findings, we conducted a replication study using the 2002 ISSP data (ISSP Research Group, 2013), which contain five identical and two similar gender ideology items and seven of the eight countries that we examine. The results show similar class structures and sizes, which indicates that the results are robust to estimation in a different sample (the replication study is available from the authors upon request).

## Findings

The results generated by LCA are the prevalence (or size) of the five ideology classes and the respondents' conditional response probabilities for each gender ideology item. The latter information was used to interpret the meaning of the different classes and to label them accordingly in line with our theoretical framework and the state of research. The labels thus offer a way of interpreting our findings, although we acknowledge that our interpretation is limited by the few ideology items available in our data set. In particular, we cannot capture the full range of connotations associated with these concepts in the qualitative literature.

In addition to the two unidimensional classes based on the separate spheres framework, egalitarian (joint spheres responses on all items) and traditional (separate spheres responses on all items), our model revealed three classes that combined beliefs in joint and separate spheres in various multidimensional ways. These comprised the classes we labeled *intensive parenting*, *egalitarian essentialism,* and *moderate traditional*. To assess country and sex differences, we calculated the predicted probabilities of class membership for men and women per country.

Table 2 presents the conditional probabilities of a joint spheres (egalitarian) response in each class, whereas Appendix Figure A4 provides a graphic representation of the same data. Table 3 displays predicted probabilities of class membership by sex and country. The findings are presented by class (not according to the hypotheses, which will be discussed in depth in the discussion section).

**Table 3:** Predicted Probability of Class Membership by Country and Sex

		Class label				
		Egalitarian	Egalitarian essentialism	Intensive parenting	Moderate traditional	Traditional
Class size overall, %		42.8	22.3	22.0	7.3	5.6
Country						
Czech Republic	Women	.45	.32	.15	.07	.01
	Men	.35	.38	.17	.09	.02
Western Germany	Women	.52	.15	.15	.08	.09
	Men	.41	.19	.18	.11	.12
Italy	Women	.49	.13	.29	.06	.03
	Men	.38	.16	.33	.09	.05
Netherlands	Women	.60	.11	.10	.16	.02
	Men	.48	.14	.12	.23	.03
Poland	Women	.26	.31	.33	.05	.05
	Men	.18	.34	.35	.06	.06
Spain	Women	.50	.30	.16	.01	.03
	Men	.39	.37	.18	.02	.04
Switzerland	Women	.32	.30	.25	.07	.06
	Men	.23	.34	.27	.08	.07
Sweden	Women	.79	.14	.04	.02	.01
	Men	.70	.20	.05	.03	.02

*Source*. European Values Study (2011), multinomial logit model, calculations by authors.

*Note*. Data weighted by population size. Predicted probabilities sum up horizontally to 1, corresponding with 100%.

### *Egalitarian Class*

The biggest class in our sample of eight European countries, comprising 42.8% of respondents, corresponded to the ideology class we have labeled *egalitarian*. Respondents in the egalitarian class consistently had the highest conditional probability of agreeing with statements representing joint spheres of earning and caring. The probability of class membership in the egalitarian class was highest among Swedes, followed by Dutch, western Germans, and Spanish respondents. The prevalence of the egalitarian class was lowest in Poland and Switzerland. Sex differences were very pronounced in all countries, with women being substantially more likely to fall into the egalitarian class (the difference ranged from 8% in Poland to 12% in the Netherlands).

### *Egalitarian Essentialist Class*

The second class, capturing 22.3% of respondents, endorsed female homemaking but believed in joint spheres for all other items, including the acceptance of maternal employment. We believed this class corresponded with the empirical pattern described in earlier research as *egalitarian essentialism* (Charles & Grusky, 2004; Cotter et al., 2011). The estimated probability of members in this class giving a joint spheres response to the statements “A job is alright, but what most women really want is a home and children” and “Being a housewife is as fulfilling as working for pay” was very low at 28% and 12%, respectively. This was contrary to the high probability of a joint spheres response for all other items. This support for female homemaking and maternal employment reflected an egalitarian essentialist notion of choice, specifically, that women may choose whether to work or be homemakers. The egalitarian essentialism class was most prevalent in the Czech Republic, Poland, Spain, and Switzerland, covering between 30% (Spanish women) and 38% (Czech men) of the sample. The probability of falling into this class was between 11% and 20% for all other countries, suggesting a relatively wide diffusion. The sex differences, although consistently showing men having a higher probability of being in this class, were relatively small (between 3% and 7%).

### *Intensive Parenting Class*

We labeled the third class, which captured 22% of respondents, *intensive parenting*. This class appeared to prioritize family over paid work by disapproving of maternal employment, yet at the same time believing that men should contribute to care and household work. Members in this group had a low probability of agreeing that working mothers can have just as warm a relationship with their children as stay‐at‐home mothers and consistently believed that a preschool child suffered when the mother was employed (the estimated probability of a joint spheres response to this statement was as low as 0.01%). At the same time, members in this class were likely to believe that men and women should contribute to the household income (91%), that men should take the same responsibility for household and children (98%), and that men are as suited to care for children as mothers (76%). The strong claim of father's involvement in unpaid work and women's equal responsibility for the household income in this class was noteworthy becauses it appeared to conflict with the rejection of maternal employment. This contradiction was reflected in the intermediate levels of acceptance of the housewife ideal, whereby 62% and 39% gave a joint spheres response to the housewife role being fulfilling and women really wanting a home and children, respectively. This mix of respondents' beliefs in joint and separate gendered spheres points, in our view, to intensive parenting*,* whereby individuals support a child‐centered approach to parenting for fathers, but especially for mothers of young children. Membership in the intensive parenting class was most prevalent in Italy, Poland, and Switzerland. Respondents from the Netherlands and Sweden were the least likely to be part of this class. Sex differences were consistently small (they were largest in Italy, where men were 4% more likely to be part of this class).

### *Moderate Traditional Class*

The final two classes, which we labeled *moderate traditional* and *traditional*, were rather small, capturing 7.3% and 5.6% of respondents, respectively. The class we labeled *moderate traditional* was characterized by no distinct beliefs in either joint or separate spheres regarding working mothers and the housewife ideal (roughly equal to the overall mean in the sample), but a low level of confidence in men's caring and domestic capabilities. The probability that members of the moderate traditional class agreed that fathers were suited as caregivers was as low as 28%; similarly, only 30% agreed that men should take the same responsibility for the home and children. Unlike the other classes, we did not anticipate finding this class based on the state of research. Indeed, this class turned out to be predominantly a country‐specific profile, as membership in this class was highest in the Netherlands, comprising 23% of Dutch men and 16% of Dutch women, and low in the other countries. The sex differences in all countries other than the Netherlands were negligible.

### *Traditional Class*

Finally, the class best reflecting traditional gender ideologies (5.6% of the sample) consisted of respondents with consistently low relative probabilities of believing in joint spheres. All estimated probabilities of agreeing with egalitarian statements were below 50%, with the exception of a 72% probability of agreeing with men's equal responsibility for home and children, which was still distinctly below the sample mean of 92% for this item. The prevalence of this class was highest among men from western Germany (12%) and Switzerland (7%). Sex differences were generally small, but were the largest for western Germany, with 3% more men than women in this class. It is important to note that although this class had the most consistent beliefs in separate spheres of all the classes identified in our analysis, members of this class still showed a tendency toward joint spheres for women's contribution to the household income and father's participation in care and domestic work. This suggests that there was no class in our sample that displayed an unwavering belief in separate spheres.

## Discussion

In this article, we aimed to assess (1) whether gender ideologies are unidimensional, as has long been theorized and operationalized in quantitative research, or rather multidimensional, as is indicated by more recent research; (2) whether gender ideologies vary across countries and between men and women; and (3) whether this variation is associated with country‐specific work–family policy settings. This was accomplished by drawing on comparative work–family policy research (Saraceno & Keck, 2011) and normative policy feedback (Campbell, 2012; Gangl & Ziefle, 2015). We applied a LCA to allow for multidimensional gender ideology profiles within and between countries. We first discuss our findings in light of our empirical expectations, then move to drawing wider theoretical conclusions, and finally highlight the methodological contributions and shortcomings of this study.

According to our conceptual framework and in line with recent research, we expected to find more than one dominant gender ideology in Europe, including both unidimensional and multidimensional ideologies (Hypothesis 1). This expectation was clearly supported by our analysis and fits recent empirical assessments of multidimensional gender ideologies in cross‐national research (Knight & Brinton, 2017; Yu & Lee, 2013). Overall, in our sample of eight European countries, multidimensional gender ideologies covered more than half of the respondents. This empirical prevalence of multidimensional ideologies, in particular, of egalitarian essentialism and intensive parenting, highlights the salience of our suggested framework, as it allows both unidimensional and multidimensional ideologies to be conceptualized and assessed. Importantly, we found no ideology profile that represents a consistent adherence to separate spheres, suggesting that beliefs in the full spectrum of separate spheres no longer exist in modern‐day Europe. This provides strong confirmation to our argument regarding the inadequacy of the unidimensional egalitarian or traditional axis.

Within our suggested framework and based on the interest and experience related ideology mechanisms suggested by Bolzendahl and Myers (2004), we expected egalitarian ideologies to be more widespread among women and traditional ideologies to be more widespread among men (Hypothesis 2a). This hypothesis was only partly supported by our findings. In line with our expectations, egalitarian ideologies were substantially more widespread among women than men, with differences ranging from 8% (Poland) to 12% (the Netherlands). Support for traditional ideologies, however, was very low throughout (only 5.6% of our sample), and although more men than women agreed with traditional statements within each country, gender differences were relatively small (below 3%). Given the low prevalence of the traditional class, any apparent gender differences need to be treated with caution.

Similarly, our second gender‐based hypothesis, that gender differences would be less pronounced within the multidimensional ideologies than within the unidimensional ideologies (Hypothesis 2b), was also only partially supported. Although men in any given country consistently supported each multidimensional gender ideology more than women, the gender differences were generally small. In particular, they were consistently much smaller in magnitude when compared with those found for the egalitarian profile, but they were in some instances larger than those found for the traditional profile. Support for egalitarian essentialism, for instance, was 6% to 7% higher among men than women in the Czech Republic, Spain, and Sweden. Support for intensive parenting was less gendered (with a 1% to 4% difference between women and men), with the greatest difference being in Italy (4%). Finally, moderate traditional ideologies were rare among both men and women throughout countries, except for the Netherlands, where 7% more men than women in our sample constituted this ideology group. All other gender differences were generally small to negligible (less than 3%). These findings provide no clear‐cut corroboration or challenge to the argument that interest‐based mechanisms are a driver of gender differences in ideologies (cf. Connell, 2005; Gregory & Milner, 2011). One one hand, it could be argued that men supporting egalitarian essentialism and intensive parenting embrace joint caring and thus the greater involvement of men in the domestic sphere. On the other hand, these two multidimensional ideologies differ from egalitarian ideologies mostly with respect to women's roles, not men's. In particular, both egalitarian essentialism and intensive parenting are less favorable toward maternal employment. This further highlights that multidimensional ideologies are more ambivalent with respect to whether men or women benefit more from the corresponding divisions of labor. Future research on the individual fit between gender ideologies and behavior, specifically, participation in joint or separate spheres, might shed more light on the role of the experienced‐based mechanism suggested by Bolzendahl and Myers (2004).

Our third expectation (Hypothesis 3) was that the prevalence of particular gender ideologies would vary across national work–family policy settings. This expectation was based on the assessment that the work–family policies adopted in European welfare states could reflect either unidimensional or multidimensional policy solutions to families' needs for earning and caring. Linking this to policy feedback theory, we expected that how consistently (or abruptly) these policy settings had developed over time would influence the dispersal of different gender ideology classes across our sample of countries. Our hypothesis was confirmed: The prevalence of the gender ideology profiles did indeed vary across national settings. After applying our analytical framework to the work–family policy settings in each of the eight countries under study, we formulated specific subhypotheses.

For Sweden, the country in our sample with the most consistent policy path supporting joint spheres of earning and caring, we expected egalitarian gender ideologies to prevail and to be more widespread than in other countries (Hypothesis 3a). In line with our expectation, support for egalitarian ideologies was indeed markedly higher in Sweden when compared with the other countries, and most Swedish women (79%) and Swedish men (70%) in our sample belonged to the egalitarian ideology class. Support for other ideologies was low. For Swedes holding egalitarian ideologies, correspondence between gender ideology and work–family policies was high.

Given that no countries in our sample consistently promoted separate spheres, we did not expect any countries to show a distinct traditional ideology (Hypothesis 3b). Our analysis confirmed this hypothesis. The prevalence of the traditional profile was consistently low in all countries, with the highest incidence among western German men (12%).

For Germany, a country that had just recently adopted a work–family policy resembling the Swedish model when our data were collected, we expected the strong emergence of egalitarian ideologies to compete with both traditional and multidimensional ideologies (Hypothesis 3c). This expectation was supported by our data. In line with the comparatively generous paid parental leave and the paid share reserved for fathers, egalitarian ideologies were widespread in the western part of Germany (52% of women and 41% of men), as were ideologies of egalitarian essentialism and intensive parenting (each cluster covers almost 20% of men and 15% of women). Germans also had the highest probability of being in the traditional class when compared with the other countries in our sample. The latter findings correspond to the fact that the German policy setting, although effectively encouraging joint caring since 2007, continued to offer high levels of institutional support for separate spheres.

Unlike our expectation that multidimensional gender ideologies would prevail in the remaining countries (Hypothesis 3d), all of which offered limited work–family policy support for joint earning and caring, egalitarian ideologies prevailed in the Netherlands (60% of women and 48% of men) and to a lesser extent also in Spain (48% of women and 38% of men), Italy (49% of women and 38% of men), and the Czech Republic (45% of women and 35% of men). This signifies a spread of egalitarian ideologies even under work–family policy conditions that have been argued to be ineffective in strengthening opportunities for joint earning and caring (Ray et al., 2010). In light of the norm‐setting and role‐exposure mechanisms offered in the framework of policy feedback theory, our findings suggest that even if policies do not effectively change role exposure, the norm‐setting function of work–family policies that legally enable joint spheres is potentially powerful. For example, even if parental leave, in particular the share reserved for fathers, is unpaid and thus rarely used, a preference for joint caring can be established. In addition, our findings indicate that the climate for more consequent policy changes in the direction of joint spheres would be timely in these countries. Despite the prevalence of egalitarian ideologies, it is important to note that multidimensional ideologies were nonetheless widespread in the Netherlands, Spain, Italy, and the Czech Republic. This signifies that egalitarian family models were not ubiquitous among these populations.

Finally, Poland and Switzerland confirmed our expectation of prevalent multidimensional gender ideologies. In Poland, 72% of respondents associated with one of the three multidimensional profiles, most notably egalitarian essentialism and intensive parenting; in Switzerland the share was 66%. Respondents in both countries were spread rather evenly between egalitarianism, egalitarian essentialism, and intensive parenting, whereas the prevalence of moderate traditional and traditional ideologies was low. These findings specifically indicate a lack of consensus in society, which is also reflected in the lack of policies normatively supporting either joint or separate spheres.

### *Implications*

We have motivated the broader significance of this research by arguing that the prevalence of competing gender ideologies in a country might account for the observed variation in how work–family policies impact individual behavior (Budig et al., 2012; Kremer, 2007; Pfau‐Effinger, 2005). Our findings clearly show, with the exception of Sweden, that all countries examined exhibited competing gender ideologies. In these contexts and as long as large shares of society remain divided on issues of gender, existing policies will unavoidably reflect low levels of congruence between gender ideologies and work–family policies. Future research should assess how adherence to certain gender ideologies affects practices of joint or separate gendered spheres in these contexts. Considering the potential impact of existing work–family policies by means of role exposure and norm setting, our findings suggest that each mechanism potentially promotes the formation of gender ideologies in society.

These findings are of particular relevance for scholars studying the gender revolution in European societies, which at this point is considered incomplete despite the extension of work–family policies (Dieckhoff, Gash, Mertens, & Gordo, 2016; Goldscheider et al., 2015; Sayer, 2010). The analytic approach developed in this article highlights the fact that the work–family policy setting needs to support joint spheres in both domains, earning and caring, to effectively promote the completion of the gender revolution. The enactment of policies that support joint care but not joint earning (or vice versa) is unlikely to foster more gender equitable societies and potentially promote multidimensional gender ideologies. In particular, our findings highlight the potential for further change in the direction of the gender revolution in terms of, first, paternal participation in the domestic sphere (see also Goldscheider et al., 2015) and, second, the acceptance of maternal employment. First, our finding that multidimensional ideologies have largely replaced traditional ideologies shows that a greater involvement of men in both child rearing and the home is desired and that this is the case for both men and women. This finding was essentially ubiquitous within our sample (ideologies with high agreement toward men's participation in the domestic sphere covered 87% of our sample). This is also the case for women's contribution to the household income (also 87% of our sample). Second, the primary differences between these ideologies lie in beliefs regarding mothers' labor force attachment (whereby the egalitarian profile was the only one that consistently supported maternal employment).

### *Conclusion*

In sum, our findings show clear evidence that gender ideologies are multidimensional and contain ideology profiles that are in part consistent and partly inconsistent with the separate spheres framework. Egalitarian ideologies, which consistently reflect agreement with statements representing joint spheres of earning and caring, were most widespread. The multidimensional profiles we identified were also prevalent and fit the recent descriptions of intensive parenting and egalitarian essentialism (Charles & Grusky, 2004; Faircloth, 2014b). The two traditional gender ideologies that we identified were found to be rare, covering merely 7.3% (moderately traditional) and 5.6% (traditional) of respondents. All of the gender ideology profiles occurred across the eight countries we have studied, suggesting that these ideologies, although notably varying in their prevalence, go beyond national borders or particular policies. The majority of countries had multiple predominant gender ideology profiles—and these were not necessarily compatible. This is in line with the gender culture approach, which contends that several dominant ideologies may coexist (Pfau‐Effinger, 2012; Pfau‐Effinger & Euler, 2014). In addition, our research highlights the fact that in these contexts, existing policies support the ideologies held by some groups, but not others.

### *Methodological Contributions*

Although it has been argued that alternative types of measurement need to be devised to improve the measurement of gender ideologies (Davis & Greenstein, 2009, p. 99), we demonstrate that by changing the conceptualization and operationalization of gender ideology the old measures become more reflective of actual societal trends. Although we acknowledge that this study has some limitations (which are discussed next), the fact that four of the five prevalent ideology profiles that we found mirror expectations based on recent qualitative and quantitative research is reassuring and points to the advances that can still be made using the old and arguably outdated gender ideology measures.

### *Limitations*

There are a number of limitations to this research. First, we use only eight European countries in this exploratory analysis. The benefit of this strategy is that it enabled us to assess cross‐country variation without losing sight of the particular work–family policies at play in each country and their development over time. The drawback of our eight‐country approach is that we can only make limited inferences about other countries. Second, our analyses are not able to establish any causal links between work–family policies and gender ideologies, as we are not able to use policies as explanatory variables to directly predict class structures. Moreover, as gender ideologies are believed to change over time, both as individuals mature and as historical time passes (Davis & Greenstein, 2009, p. 95), future research should examine how the gender ideology classes have changed since 2008 (the most recent year of EVS data collection at the time of publication). Third, our investigation of the gender ideologies identified in the (primarily qualitative) literature is of course limited by the few ideology items available in our data set. This implies that we are not able to fully grasp the deeper meanings that concepts such as intensive parenting or egalitarian essentialism undoubtedly have (e.g., the importance of parent's constant intellectual stimulation of the child, as emphasized by Wall, 2010). We merely offer interpretations of our findings in light of the state of theorizing. Still, the fact that our data and interpretation correspond with other quantitative studies using partly similar (Cotter et al., 2011) and partly more elaborate items to address these concepts (i.e., Liss et al., 2013; Schiffrin et al., 2014) make us confident that our approach is fruitful and will stimulate future research aiming at improving the measurement of gender ideologies.

In conclusion, our analyses provide strong support for our suggested framework that both gender ideologies and work–family policies need to be conceptualized as being multidimensional. First, we show that a unidimensional operationalization of egalitarian versus traditional ideologies has in fact become obsolete and point to the need to conceptualize gender ideologies as multidimensional within the framework of gender separate or joint spheres. Second, we find clear evidence of distinct but widespread gender ideology profiles throughout all eight of the European countries studied. In particular, our research provides empirical evidence that egalitarian essentialism and intensive parenting have become widespread ideologies, which have replaced more traditional gender ideologies. Third, we find varying degrees of congruence between the structure and configuration of national gender ideology profiles and work–family policies. This leads us to conclude that the diverse, and frequently conflicting, gender ideology profiles within most countries place strong limits on the capacity of policies to adequately address these multiple orientations. We argue that this, together with the frequent operationalization of gender ideology as being unidimensional, underlies why so much previous research is unable to find a clear link between gender ideology and policy frameworks. Our findings stress the importance that both national and cross‐national research be sensitive to within‐country variation in gender ideologies.

## Note

The research leading to these results has received funding from the European Research Council (ERC) under the European Union's Seventh Framework Programme (FP/2007‐2013)/ERC Grant Agreement No. 263651, Principal investigator: Daniela Grunow.

## Supporting information

Appendix S1. Gender Ideologies in Europe: A Multidimensional Framework
