# Supplementary material for: Gender Ideologies in Europe: A Multidimensional Framework
Source: J Marriage Fam. 2018 Jan 11;80(1):42–60. doi: 10.1111/jomf.12453 (PMC5817238; doi:10.1111/jomf.12453)
Supplement: Supplementary file 1 — Appendix S1. Gender Ideologies in Europe: A Multidimensional Framework [file JOMF-80-42-s001.docx]

**Gender Ideologies in Europe: A Multidimensional Framework**

APPENDIX [to be published online]

Table A1. *Overview of work-family policies (2008)*

|  | Parental Leave | | Childcare | | Promotion of Fathers as Caregivers | | Maternity Leave | |
| --- | --- | --- | --- | --- | --- | --- | --- | --- |
|  | Duration of Parental Leave (months) | Length of payment (months) ^a^ | Childcare Usage, < age 3 (%) | Full-time childcare usage, < age 3 (%) | Paid Paternity Leave (days) | Parental Leave reserved for father (Months) | Duration (weeks) | Level of Compensation (% earned income) |
| Czech Rep. | 30.5 | 30.5 | 3 | 0 | 0 | 0 | 28 | 90 |
| Germany | 34.2 | 12.2 | 19 | 12 | 0 | 2 | 14 | 100 |
| Italy | 11 | 6 | 25 | 16 | 0 | 6 | 20 | 80 |
| Netherlands | 6 | 0^a^ | 49 | 6 | 2 | 3 | 16 | 100 |
| Poland | 36 | 0 | 2 | 2 | 0 | 0 | 18 | 100 |
| Spain | 34.5 | 0 | 36 | 18 | 14 | 0 | 16 | 100 |
| Sweden | 16 | 13.8 | 63 | 37 | 10 | 2 | 8.6 | 78 |
| Switzerland | 0 | 0 | 33^b^ | 25^c^ | 0 | 0 | 14 | 80 |

*Source* (for all countries, excluding Switzerland): Multilinks Database for 2009 (Keck, Hessel, & Saraceno, 2009). Source for Switzerland: OECD Family Database. *Notes:* ^a^The length of payment has been defined as the “[t]ime of paid parental leave multiplied by the income replacement rate of the parental leave benefit or alternatively the child rearing allowance” (Keck et al., 2009, p. 42). ^b^Collective labor agreements in the public sector (ca 20% of Dutch employees): usually 3 months of 65% paid leave. ^c^Data refer to 2009. ^d^Data refer to 2013.

Table A2. *Sample size and weights*

|  | Inhabitants 2008 | % of total | Sample size EVS 2008 | % in sample | Weight |
| --- | --- | --- | --- | --- | --- |
| Czech Republic | 10,425,000 | 0.04 | 839 | 16.16 | 0.25 |
| Germany West | 69,037,300 | 0.27 | 487 | 9.38 | 2.87 |
| Italy | 59,832,000 | 0.23 | 720 | 13.86 | 1.68 |
| Netherlands | 16,446,000 | 0.06 | 503 | 9.69 | 0.66 |
| Poland | 38,126,000 | 0.15 | 795 | 15.31 | 0.97 |
| Spain | 45,668,000 | 0.18 | 786 | 15.14 | 1.18 |
| Sweden | 9,220,000 | 0.04 | 491 | 9.46 | 0.38 |
| Switzerland | 7,701,900 | 0.03 | 572 | 11.01 | 0.27 |
| Total | 256,456,200 | 1.00 | 5,193 | 100 | 1.00 |

*Source:* EVS 2008, calculations by authors.

Table A3. *Model fit statistics*

| Classes | BIC | Lo-Mendell-Rubin LRT k-1 vs. k classes | p-value | Entropy |
| --- | --- | --- | --- | --- |
| 1 | 60464.00 | n.a. |  | n.a. |
| 2 | 35361.13 | 2243.86 | .00 | 0.62 |
| 3 | 34923.12 | 570.59 | .00 | 0.66 |
| 4 | 34646.97 | 409.91 | .00 | 0.71 |
| 5 | 34447.00 | 334.43 | .00 | 0.72 |
| 6 | 34397.64 | 195.01 | .72 | 0.74 |
| 7 | 34402.20 | 131.29 | .20 | 0.75 |
| 5.1^a^ | 34225.57 | 195.678 | .83 | 0.64 |

*Source:* EVS 2008, multinomial logit model, calculations by authors. *Notes:* Data weighted by population size. ^a^Model including significant direct effects of covariates on latent class indicators

FIGURE A4. GRAPHIC REPRESENTATION OF THE ESTIMATED PROBABILITY OF AGREEMENT WITH STATEMENTS PER CLASS
